# Shikonin improves the effectiveness of PD-1 blockade in colorectal cancer by enhancing immunogenicity via Hsp70 upregulation

**DOI:** 10.1007/s11033-023-09056-2

**Published:** 2024-01-06

**Authors:** Jinghua Chen, Jie Liu, Xiaolin Liu, Jun Wang, Xiumei Wang, Xin Ye, Qi Xie, Jing Liang, Yan Li

**Affiliations:** 1 Shandong Provincial Qianfoshan Hospital, Shandong University, Jinan, China; 2 Shandong Key Laboratory of Rheumatic Disease and Translational Medicine, Department of Oncology, Shandong Lung Cancer Institute, The First Affiliated Hospital of Shandong First Medical University & Shandong Provincial Qianfoshan Hospital, Jinan, China; 3 Department of Pediatric Intensive Care Unit, Shandong Provincial Maternal and Child Health Care Hospital Affiliated to Qingdao University, Jinan, China; 4 Department of Oncology, The Yuncheng Chenxin Hospital, Heze, China

**Keywords:** Shikonin, PD-1 blockade, Colorectal cancer, Hsp70, Stable microsatellites

## Abstract

**Background:**

PD-1 blockade has shown impressive clinical outcomes in colorectal cancers patients with high microsatellite instability (MSI-H). However, the majority of patients with colorectal cancer who present low microsatellite instability (MSI-L) or stable microsatellites (MSS) show little response to PD-1 blockade therapy. Here, we have demonstrated that Shikonin (SK) could induce cell death of CT26 cells via classically programmed and immunogenic pathways.

**Methods and results:**

SK promoted the membrane exposure of calreticulin and upregulated the expression of heat shock protein 70 (Hsp70). The upregulation of Hsp70 was dependent on ROS induced by SK and silencing of PKM2 in CT26 cells reverts ROS upregulation. Besides, SK synergizes with PD-1 blockade in CT26 tumor mice model, with the increase of intramural DC cells and CD8^+ ^T cells. The expression of Hsp70 in tumor tissue was also increased in combinational SK plus αPD-1 therapy group.

**Conclusions:**

Our study elucidated the potential role of ‘Shikonin-PKM2-ROS-Hsp70’ axis in the promotion of efficacy of PD-1 blockade in CRC treatments, providing a potential strategy and targets for improving the efficacy of PD-1 blockade in colorectal cancer.

## Introduction

Colorectal cancer (CRC) is the most common digestive tract tumor, accounting for approximately 9.7% of all cancer patients and approximately 8.5% of tumor-related deaths [1]. The standard treatment for metastatic CRC is chemotherapy combined with targeted therapy, such as anti-angiogenic agents (ramucirumab, bevacizumab or aflibercept) or anti-EGFR drugs (cetuximab or panitumumab) for patients with RAS wild-type [2, 3]. However, in cases where these common treatments fail, treatment options are limited. In recent years, immunotherapy for cancer has made great progress. Among these advances, PD-1 blockade has elicited promising clinical response in a variety of solid tumors, including melanoma and non-small cell lung cancer [4, 5]. However, only 5% patients with microsatellite instability-high (MSI-H)/deficient mismatch repair (dMMR) CRC could benefit from PD-1 blockade therapy [6, 7]. For the larger subgroup of non-MSI-H/dMMR CRC patients, combined regimens are strongly needed and might be an ideal strategy to address the conundrum [8].

Damage-associated molecular patterns (DAMPs) are endogenous molecules released from damaged or dying tumor cells, and they have been shown to activate the innate immune system by interacting with innate receptors [9]. DAMPs could modulate the tumor immune microenvironment (TIME) and influence tumor growth. Immunogenic cell death (ICD), is a form of cell death which activates adaptive immunity and induces long-term immune memory [10]. ICD could be induced by endoplasmic reticulum (ER) stress and reactive oxygen species (ROS) generation, which leads to release of DAMPs [11–13].

Shikonin (SK) is the bioactive purplish red naphthoquinone extracted from the natural plant Zongfu root [14]. Some researchers have found that SK could induce apoptosis of colon cancer cells, and regulate invasion and autophagy [15, 16]. SK has also been proved to be an inducer of ROS in tumor cells, whereby inducing apoptosis and release of DAMPs [17]. Lin et al. has found that Shikonin can enhance the cellular immunogenicity of tumor vaccines through different DAMPs. Among three DAMPs tested, they found that the Hsp70 is the most important component in facilitating DC immunity on inhibiting metastasis of mouse tumors and prolonging mouse survival [18]. However, the complete understanding of the mechanism behind Hsp70 upregulation induced by Shikonin is still under investigation. Chen et al. has found that SK could induce ICD of melanoma B16 cells and enhance function of dendritic cells (DCs) [19]. Previous studies have confirmed that SK and BET inhibitor JQ1 can synergistically exert anti-tumor effects by reshaping the tumor immune microenvironment [20]. However, there is limited research available on the effects of combining shikonin (SK) and PD-1 blockade for the treatment of CRC. Further studies are required to explore the potential benefits and mechanisms of this combination therapy. In the present study, we investigated the effect of SK combined with αPD-1 therapy in CT26 tumor model, which is known to be microsatellite stability (MSS). In addition, we investigated the synergistic mechanism of combining SK with αPD-1 by examining the effects on apoptosis, release of DAMPs, and changes in the tumor immune environment. Our study demonstrated a theoretical basis for SK combined αPD-1, providing a potential treatment strategy for CRC who could not benefit from PD-1 blockade.

## Materials and methods

### Chemicals, reagents and antibodies

Shikonin was purchased from Sigma-Aldrich (Cat No. 517-89-5, St. Louis, MO, USA). Purified anti-mouse PD-1 (clone RMP1-14) was purchased from BioXcell (Cat No. BE0146, West Lebanon, NH, USA). Fluorescent-labeled anti-mouse CD4 antibody (Cat No. 100406), anti-CD8 antibody (Cat No. 301014), anti-CD11b antibody (Cat No. 101212), anti-CD11c antibody (Cat No. 117308) were purchased from Biolegend (San Diego, CA, USA). Anti-mouse calreticulin antibody (Cat No. ab92516) and anti-mouse Hsp70 antibody (Cat No. ab181606) were purchased from Abcam (Cambridge, UK). 7-AAD Viability Staining Solution was purchased from Invitrogen (Cat No. 00-6993, Waltham, MA, USA). Acetylcysteine was purchased from MCE (Cat No. HY-B0215, NJ, USA). 2,7-Dichlorodihydrofluorescein was purchased from APExBIO (Cat No. C3890, Houston, TX, USA).

### Cell

CT26 cells were obtained from the cell bank of the Chinese Academy of Sciences (Shanghai, China) and cultured in RPMI1640 containing 10% FBS at 37 °C and 5% CO_2_ condition.

### Mice

20 SPF female BALB/c mice (6–8 weeks old, weight 16–19 g) were purchased from Beijing Vital River Laboratory Animal Technology Co., Ltd. All animal experiment has been approved by the Ethics Committee of the first affiliated Hospital of Shandong first Medical University. All mice were exposed to ambient humidity of 60% and temperature of 20–26 °C to ensure a 12 h light cycle every day.

### In vivo CT26 tumor mouse model

6 × 10^5^ CT26 cells were subcutaneously inoculated into BALB/c mice in the right inguinal region. The tumor volume was calculated as 0.5 × length × width^2^. Tumor-bearing mice were randomized into 4 groups: Group A: IgG (n = 5); Group B: IgG + SK (n = 5); Group C: anti-PD-1 mAb (n = 5); Group D: anti-PD-1 mAb + SK (n = 5). Mice were given drugs when the tumor volume grew to about 62.5 mm^3^. SK was intraperitoneally injected at 3 mg/kg every 2 days for a total of seven times [17, 21, 22], and the same volume of DMSO was given to group A and group C as control for SK. Anti-PD-1 antibody was intraperitoneally injected at 50 µg/mice on the 5th and 9th day [23, 24], and the same volume of IgG was given to group A and group B as control for αPD-1. On the 16th day, the mice were sacrificed and the volume of tumor was measured.

### Apoptosis analysis

CT26 cells seeded in six-well plates (4 × 10^5^ cells/well) were treated with DMSO, SK (5 µM or 10 µM) for 24 h, and then cells were harvested, washed twice with ice-cold PBS, stained with Alexa Fluor 488-Annexin V and 7-AAD for 15 min at room temperature in the dark, and then analyzed by using flow cytometry.

### ROS generation

CT26 cells were seeded in six-well plates (4 × 10^5^ cells/well) and treated with DMSO, SK (5 µM or 10µM), NAC (10 mM) for 24 h. NAC was given 2 h before SK treatments, and cells were incubated with 10 µM H_2_DCFDA for 40 min in 37℃ cell Incubator [25]. ROS expression in cells were analyzed by fluorescence microscope.

### CRT exposure analysis

CT26 cells seeded in six-well plates (4 × 10^5^ cells/well) were cultured with DMSO, SK (5 µM or 10 µM) for 24 h. The treated cells were collected, incubated with Alexa Fluor 488-anti mouse CRT antibody for 30 min at room temperature in the dark, and analyzed by flow cytometer to identify CRT exposure.

### CRT, HMGB1 and Hsp70 expression analysis

Tumor cell lysate samples were prepared as previously described [19]. The protein samples were resolved by SDS-PAGE using 10 or 15% stepwise gels. The resolved proteins were transferred to a PVDF membrane, and the membrane was blocked with 5% non-fat dry milk in TBST buffer for 60 min at room temperature. The membranes were incubated with primary antibodies (1:1000 dilutions) overnight at 4 °C, and then with HRP-conjugated secondary antibody (1:8000 dilutions) for 1 h at room temperature and washed with TBST buffer. The transferred proteins were visualized with an enhanced chemiluminescence (ECL) detection assay kit. Quantification of bands was performed using Image J software (National Institutes of Health, Bethesda, Md, USA).

### Transfection

According to the manufacturers’ protocol, all the small interfering RANs (siRNAs) including siRNA-pyruvate kinase M2 and negative control siRNA (si-NC) (GenePharma) were transfected into CT26 cells using the Lipofectamine 3000 Reagent (Invitrogen). The silencing effect was confirmed by western blotting.

### Statistical analysis

Each experiment was performed on at least three separate occasions and the number of independent experiments carried out are stated in figure legends. Statistical analyses were performed using SPSS 19.0 (IBM, Armonk, NY, USA). Data that conform to the normal distribution are presented as the mean ± standard deviation (SD). *t *test was used to determine the statistical significance of differences between two variables. A one-way analysis of variance (ANOVA) test was used for statistical comparisons between groups (when number of groups are more than 3). P < 0.05 was considered statistically significant. Prism version 6 (GraphPad Software Inc., San Diego, CA, USA) was used for graphic presentation.

## Results

### CT26 cell death was induced by Shikonin via classically programmed and immunogenic pathways

**Fig. 1:**
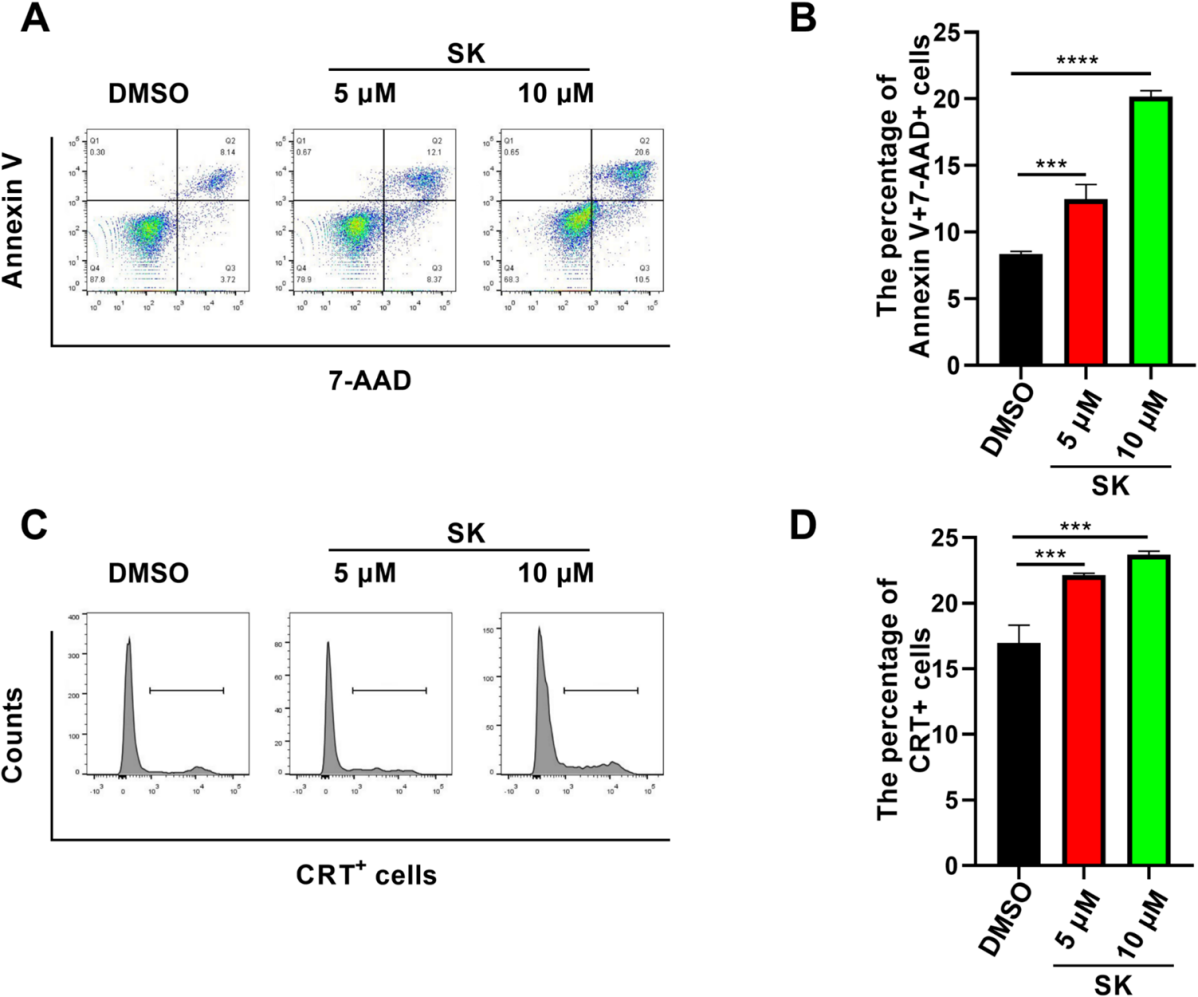
CT26 cell death was induced by Shikonin via classically programmed and immunogenic pathways. CT26 cells were treated with SK (5 μM or 10 μM) for 24 h and then cell apoptosis was determined. **A** Cells were stained by Annexin V and 7-AAD and were analyzed by flow cytometry. **B** The percentage of Annexin V+ and 7-AAD+ cells are shown. **C** CRT cell surface expression upon treatment with SK were determined by flow cytometry. **D** The percentage of CRT+ cells are shown. Data are expressed as mean ± SD of three independent experiments. ∗∗∗P＜0.001; ∗∗∗∗P＜0.0001.

**Fig. 2:**
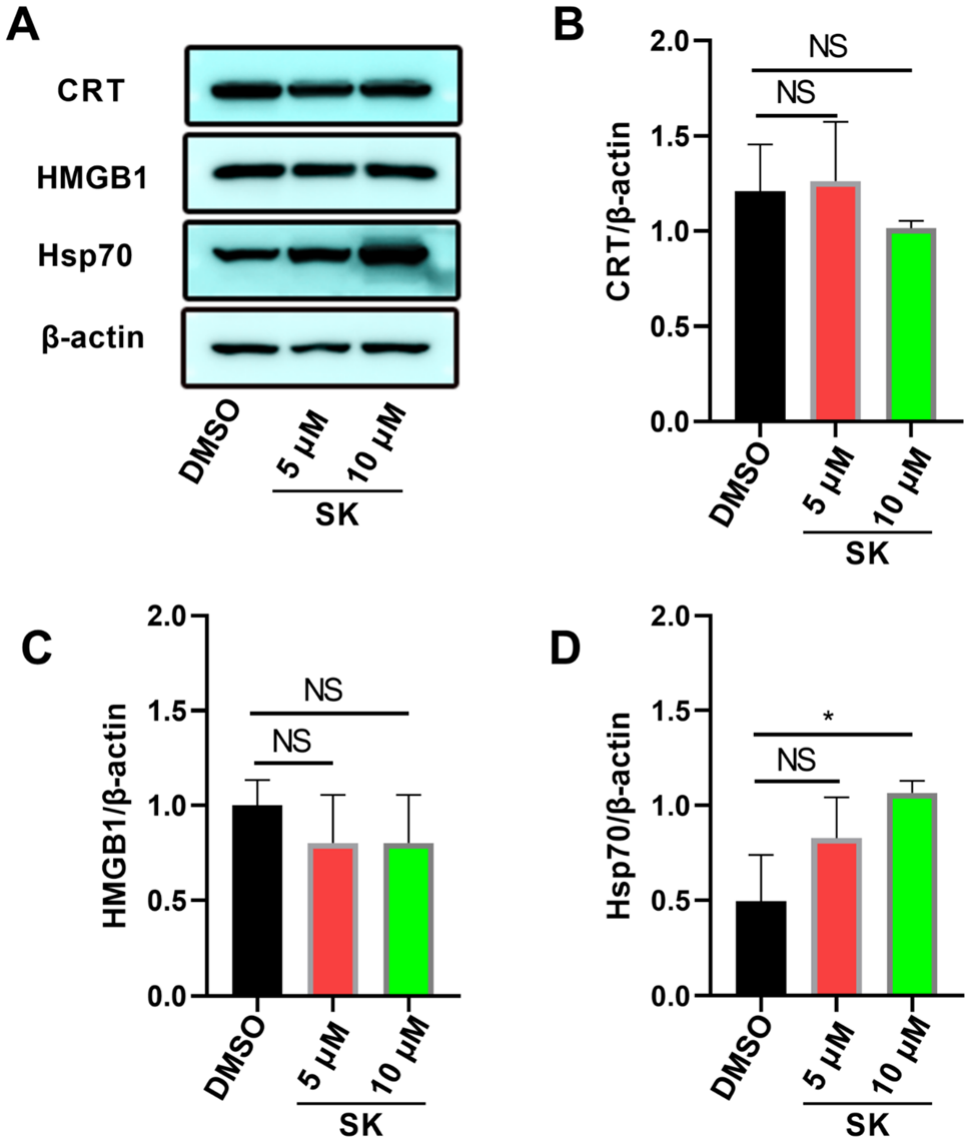
SK induced-ICD makers of CT26. CT26 cells were treated with SK (5 µM or 10 µM) for 24 h.** A** The expression of CRT, HMGB1 or Hsp70 in CT26 cells was determined using western blotting. **B**–**D** β-actin was used as a loading control and the ratio of target protein to β-actin was shown. Data are expressed as mean ± SD of three independent experiments. ∗P < 0.05

To evaluate anti-tumor effects of SK on colorectal cancer cells, CT26 cells, the murine colorectal carcinoma cell line, were treated with SK at different concentrations. The apoptosis rate of CT26 cells was detected using flow cytometry. Compared with control group (8.36 ± 0.193%), the percentage of Annexin V^+^ 7-ADD^+^ cells after SK treatments (5 µM) was increased (12.47 ± 1.097%) (P < 0.001), while further been increased to 20.17 ± 0.451% at high SK concentration (10 µM, P < 0.0001) (Fig. 1A, B), indicating that SK significantly induced apoptosis of CT26 cells in a dose-dependent manner. Meanwhile, in order to figure out whether SK induces immunogenic cell death (ICD), three DAMPs expressions on CT26 cells were determined. In terms of CRT cell-surface exposure, we found that SK at 5 µM (22.13 ± 0.153%) (P < 0.001) and 10 µM (23.70 ± 0.265%) (P < 0.001) both effectively enhance CRT cell-surface exposure compared with DMSO (16.97 ± 1.350%) (Fig. 1C, D). Furthermore, the expressions of CRT, HMGB1 and Hsp70 in CT26 cells treated with SK were quantified by western blotting. In comparison with control group, there was no significant expression change of CRT and HMGB1 after SK treatments (Fig. 2A), and the expression of soluble HMGB1 determined by ELISA was not significantly changed (Data not shown). However, SK induced upregulation of Hsp70 in CT26 cells in a dose-dependent manner (P < 0.05) (Fig. 2A, D). The inconsistent results obtained from flow cytometry and Western blotting for CRT suggest that SK only aids in the movement of CRT from its intracellular compartments to the surface of the cell, without actually increasing the overall expression of the protein. The above results indicated that SK significantly induced cell death of CT26 cells via the classically programmed and immunogenic pathway.

### Shikonin-induced Hsp70 upregulation was dependent on increased reactive oxygen species

**Fig. 3:**
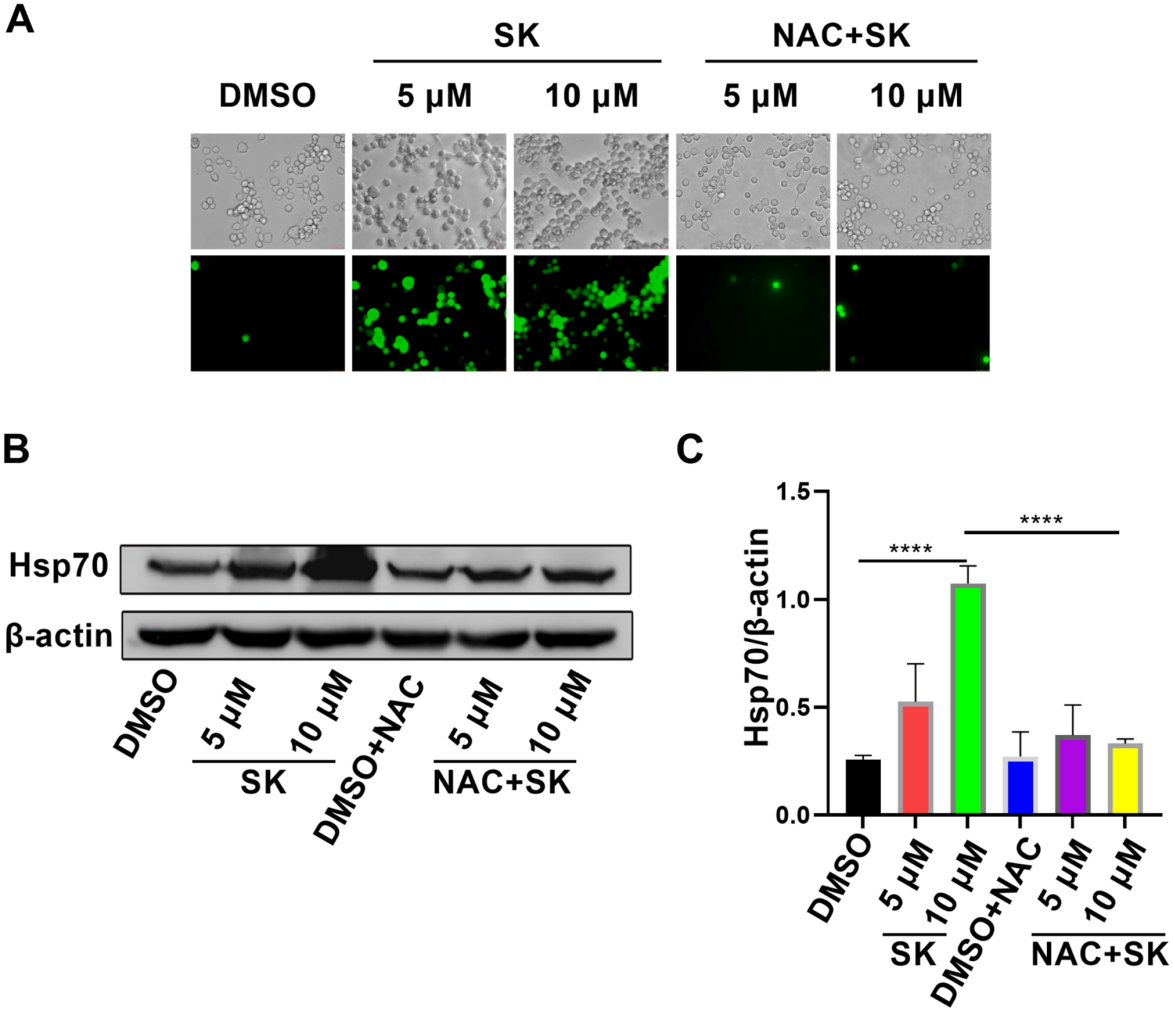
Shikonin-induced Hsp70 upregulation was dependent on increased reactive oxygen species. CT26 cells were incubated with SK (5 µM or 10 µM) or/and NAC (10 mM) for 24 h. In blocking experiments, CT26 cells were pretreated with NAC for 2 h and then stimulated with SK. **A** ROS generation in CT26 cells were indicated by the green fluorescence of 2′,7′-dichlorofluorescein (DCF) that was oxidized from 2′7′-dichlorodihydrofluorescein diacetate (H_2_DCFDA) by ROS. **B**, **C** Western blot analyses for expression of Hsp70 in CT26 cells treated with SK or/and NAC. β-actin was used as a loading control. Data are expressed as mean ± SD of three independent experiments. ∗∗∗∗P < 0.0001

Previous studies have reported that ROS can regulate the activity and expression of Hsp70 by activating certain oxidative-reduction signaling pathways [26]. To explore the potential mechanisms of Hsp70 upregulation on CT26 cells induced by SK, we investigated whether SK affect ROS production in CT26 cells. Our results showed that both SK at 5 µM and 10 µM significantly induced ROS generation in CT26 cells compared with DMSO control. In order to assure the inductive effect of ROS in the increase of Hsp70 expression, N-acetyl-L-cysteine (NAC), a ROS inhibitor, was employed in blocking experiment. As expected, NAC significantly inhibited SK-induced ROS accumulation at different concentrations (Fig. 3A). Of note, NAC significantly abolished the upregulation of Hsp70 induced by SK in CT26 cells (Fig. 3B, C), suggesting that the increase of Hsp70 expression induced by SK was dependent on SK-induced ROS in CT26 cells.

### Silencing of PKM2 in CT26 cells reverts ROS upregulation induced by Shikonin

**Fig. 4:**
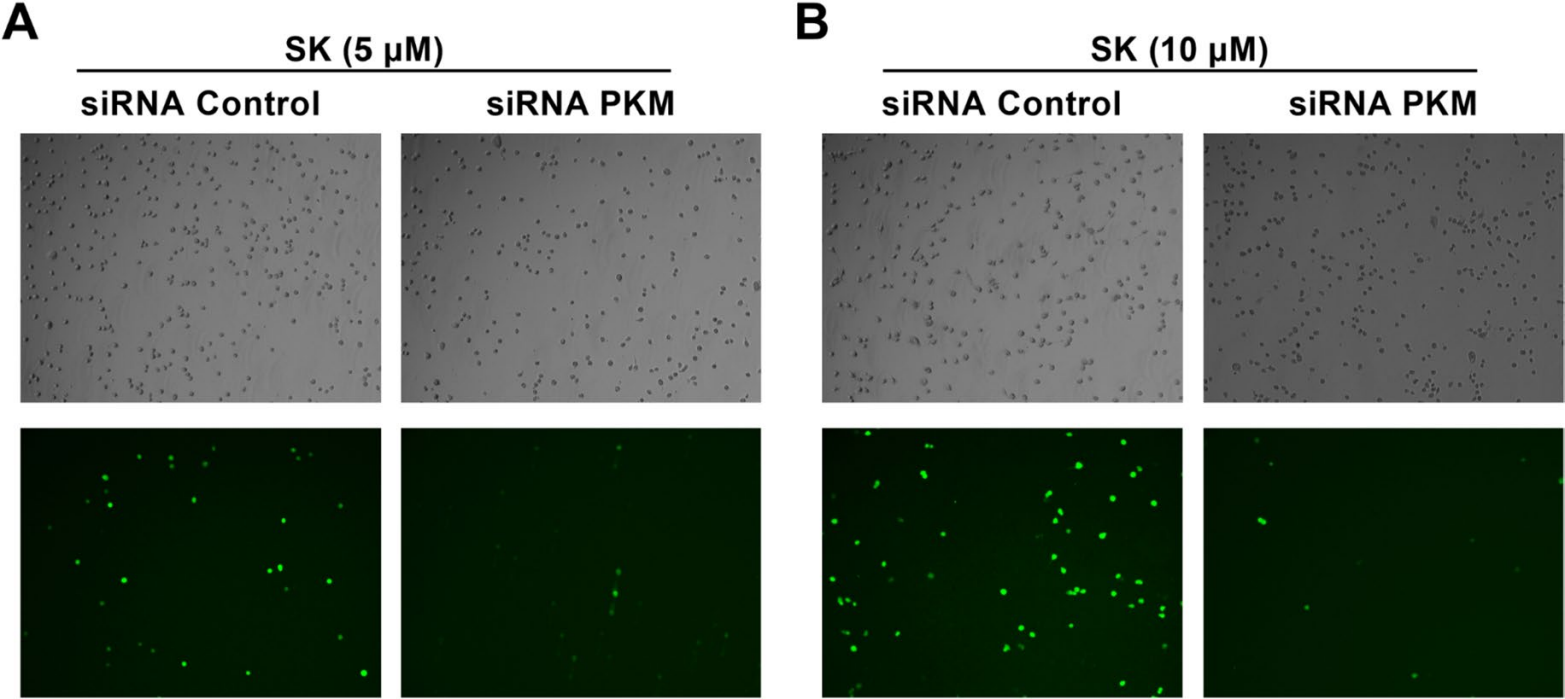
Silencing of PKM2 in CT26 cells reverts ROS upregulation induced by Shikonin. CT26 cells were transfected with a small interference oligonucleotide against PKM2 (20 µM) for 48 h, and then CT26 cells were incubated with SK at 5 µM (**A**) or 10 µM (**B**) for 12 h. The generation of ROS was determined by measuring the intensity of green fluorescence of 2′,7′-dichlorofluorescein (DCF). Data are from one of three independent experiments

It has been reported that pyruvate kinase M2 (PKM2), a glycolytic enzyme that play a critical role in aerobic glycolysis and cell growth, was increased in colorectal cancer, promoting proliferation and migration of colon cancer cells [27, 28]. Shikonin has been regarded as the effective inhibitor of PKM2 [29]. In order to clarify whether SK induced ROS in a PKM2-depentent manner, we silenced PKM2 gene expression in CT26 cells using PKM2-siRNA and determine ROS production after SK treatments. Compared with siRNA NC control, silencing of PKM2 significantly reduced the production of ROS induced by SK (Fig. 4A, B). This result indicates that increased ROS was induced by SK in a PKM2-depentent manner.

### Shikonin synergizes with PD-1 blockade in CT26 tumor mice model

**Fig. 5:**
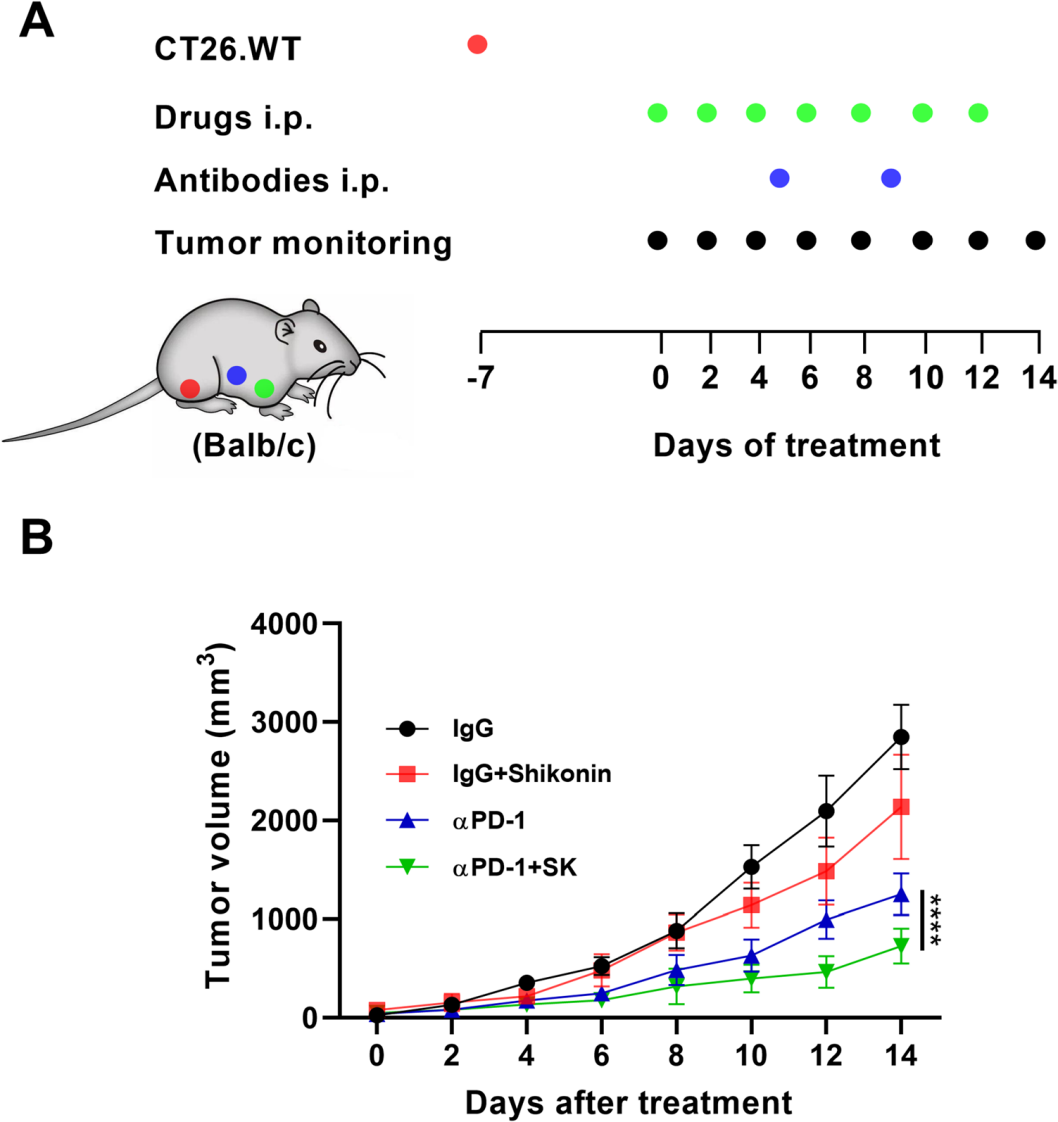
Shikonin synergizes with PD-1 blockade in CT26 tumor mice model. (**A**) Experimental design for the treatment in CT26 tumor-bearing mice. 6 × 10^5^ CT26 cells were subcutaneously inoculated into BALB/c mice in the right inguinal region. Tumor-bearing mice were randomized into 4 groups: Group A: IgG (n = 5); Group B: IgG + SK (n = 5); Group C: anti-PD-1 mAb (n = 5); Group D: anti-PD-1 mAb + SK (n = 5). Mice were given drugs when the tumor volume grew to about 62.5 mm^3^. SK or DMSO control was intraperitoneally injected at 3 mg/kg every two days for a total of seven times. Anti-PD-1 antibody or IgG control was intraperitoneally injected at 50 µg/mice on the 5th and 9th day. **B** Growth curves of CT26 tumors were measured after treatment with SK combined with αPD-1. Data are expressed as mean ± SD. ∗∗∗∗P < 0.0001

It has been reported that chemotherapy drugs could improve the efficacy of PD-1 blockade in colorectal cancer treatment by triggering DAMPs release [30]. Since SK could induce DAMPs release, we tested whether there is any synergistic effect in combinational SK and PD-1 blockade therapy. For this purpose, CT26-bearing mice were established and treated with SK, αPD-1 or SK plus αPD-1. Mice received SK and αPD-1 treatments have shown smaller tumors volume (294.1 ± 231.2mm^3^) compared to mice receiving αPD-1 monotherapy (489.2 ± 444.6mm^3^) (P < 0.0001) or IgG control group (1048 ± 1016mm^3^) (P < 0.0001) (Fig. 5), suggest that SK synergizes with PD-1 blockade in CT26 tumor mice model.

### Combinational Shikonin and PD-1 blockade modified tumor immune-microenvironment

**Fig. 6:**
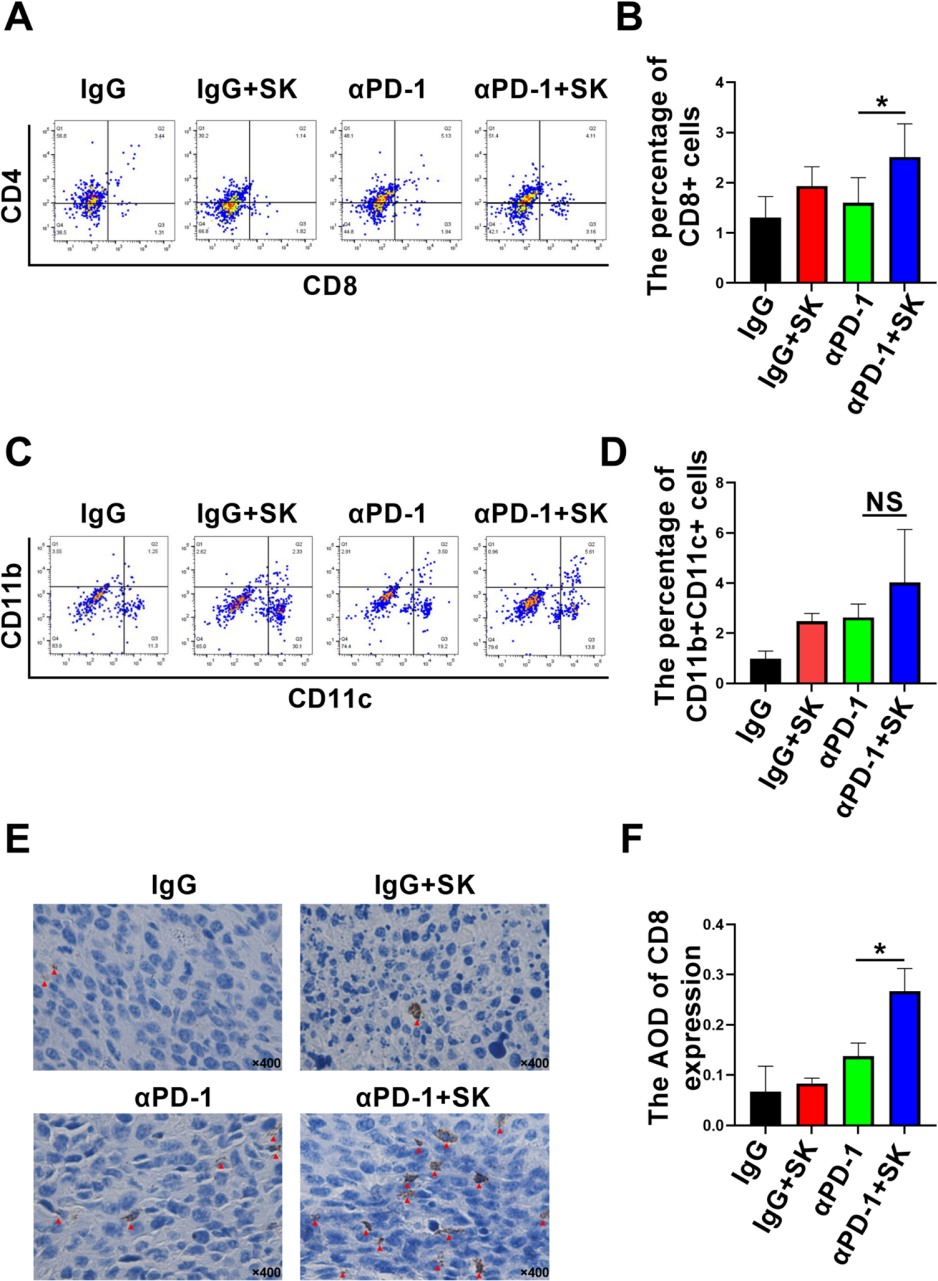
Combinational Shikonin and PD-1 blockade modified tumor immune microenvironment. Immune microenvironment in solid tumor was evaluated and the percentage of intratumoral T or DC cells were determined by using flow cytometry. The percentages of intratumoral CD8^+^ T cells (**A**, **B**) and CD11b^+^ CD11c^+^ cells (**C**, **D**) were analyzed by flow cytometry after treatments. CD8^+^ T cell in tumor was also evaluated using immunohistochemistry. **E**, **F** The average optical density (AOD) of CD8^+^ T cells was shown and data are expressed as mean ± SD. ∗P < 0.05

**Fig. 7:**
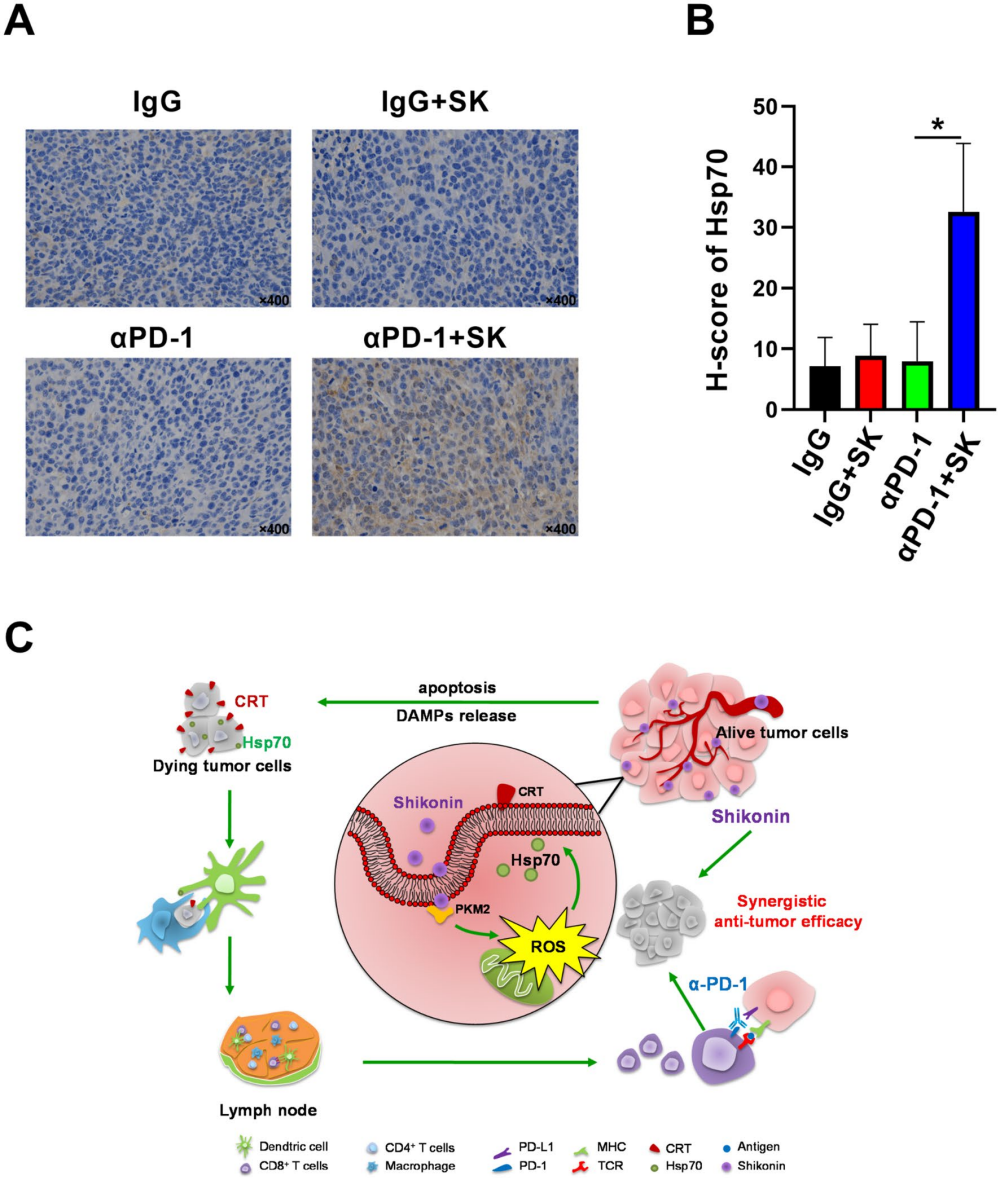
The cellular and molecular mechanism underling the synergetic effect of αPD-1 and SK in CT26 tumor mice model. **A** Hsp70 expression in tumor was determined using immunohistochemistry. **B** H-score of Hsp70 in tumor was shown and data are expressed as mean ± SD of three independent experiments. ∗P < 0.05. **C** Anti-tumor mechanism underling the synergetic effect of αPD-1 and SK. Shikonin could synergize with PD-1 blockade in CT26 tumor model, along with increased CD8^+^ T cells and DC cells. CT26 cell death was induced by SK via classically programmed and immunogenic pathways. Among three DAMPs, SK specifically induced Hsp70 upregulation dependent on increased ROS, whilst silencing of PKM2 in CT26 cells reverts ROS upregulation. Our study elucidated the potential role of ‘Shikonin-PKM2-ROS-Hsp70’ axis in the promotion of efficacy of PD-1 blockade in CRC treatments

In order to explore the cellular and molecular mechanism underling the synergetic effect of aPD-1 and SK, immune-microenvironment in solid tumor was evaluated and the percentage of DC or T cells was determined by using flow cytometry. As shown in Fig. 6, combination of SK and αPD-1 treatments significantly increased infiltration of intratumoral CD8^+^ T cells (SK + αPD-1: 2.52 ± 0.659%, αPD-1: 1.600 ± 0.501%) (Fig. 6A, B). Also combinational therapy induced a trend of increased the percentages of CD11c^+^CD11b^+^ DC cells (4.02 ± 2.112%) when compared to αPD-1 monotherapy (2.63 ± 0.538%) (Fig. 6C, D). Immunohistochemistry has been to confirm more intratumoral CD8^+^ T cells after combination of SK and αPD-1 treatments (0.27 ± 0.045) compared with IgG control (0.07 ± 0.051) (P < 0.001) or αPD-1 monotherapy (0.14 ± 0.026) (P < 0.05) (Fig. 6E, F). In addition, we found that the expression of Hsp70 in tumor tissue was significantly increased after SK and αPD-1 treatments (32.61 ± 11.260) compared with IgG control (7.14 ± 4.772) (P < 0.05) or αPD-1 alone (7.98 ± 6.519) (P < 0.05) (Fig. 7A, B). Above all, these results indicated SK and αPD-1 treatments modified tumor immune-microenvironment with the change of percentage of immune cells and expression of Hsp70.

## Discussion

In this study, we have found that Shikonin (SK) could synergize with PD-1 blockade in CT26 tumor model, along with increased DC cells and CD8^+^ T cells. CT26 cell death was induced by SK via classically programmed and immunogenic pathways. Among all DAMPs, SK specifically induced Hsp70 upregulation dependent on increased ROS, whilst silencing of PKM2 in CT26 cells reverts ROS upregulation. Our study elucidated the potential role of ‘Shikonin-PKM2-ROS-Hsp70’ axis in the promotion of efficacy of PD-1 blockade in CRC treatments (Fig. 7C).

Immune checkpoints inhibitor, αPD-1, is highly effective in patients with advanced melanoma, non-small cell lung cancer or metastatic renal cell carcinoma [31–33]. However, PD-1 blockade has been considered as the ineffective therapy in most CRC patients, except for those with MSI-H or high mutational burden. Thus, it is necessary to improve the efficacy of PD-1 blockade, and combinational therapy has been as the popular options. For the first time, we found that Shikonin could synergize with PD-1 blockade in CT26 tumor model, providing potential therapy regimen for patients with MSS CRC.

Our findings those combinational SK and PD-1 blockade modified tumor immune-microenvironment were consistent with other researchers. Lin et al. have demonstrated Shikonin effectively upregulated Hsp70 and CRT expressions, but not HMGB1 in 4T1 cells. They also found that CRT and Hsp70 mediated a critical role in Shikonin-treated 4T1 cell lysates-induced DC cell immunity, with significant CD4^+^ and CD8^+^ T cell proliferation. Besides, Hsp70 is regarded as the most critical component in enhancing DC immunity on suppressing tumor metastasis [18]. In our study, the upregulation of Hsp70 in tumor tissue was also observed after SK and PD-1 blockade treatments. Chen et al. concluded that Shikonin could effectively increase the production of specific DAMPs in B16 cells, including Hsp70, Hsp90, CRT and HMGB1. Shikonin-treated B16 cell lysates could induce DCs to a higher level of functional and phenotypic maturation, exhibiting high expression of CD86 and MHC class II and Th1 cells activation [19]. In our study, we also found that the percentage of CD11^+^CD11b^+^ DC and CD8^+^ T cells have been increased, suggesting that synergistic effect of SK with PD-1 blockade in CT26 tumor mice model might due to DC activation and cytotoxic CD8^+^ T cells infiltration.

Using the Hsp70^−/−^ mouse model, Dodd et al. proved that Hsp70 played a key role in tumor recognition by adaptive immune system and in facilitating anti-tumor immunity. Also, Hsp70^−/−^ tumors exhibits significant reduction in the infiltration of immune cells [34]. In addition, Tsang et al. found that the immunogenicity of CT26 cell was mainly dependent on the release of Hsp70 [35]. Komarova et al. also proved that Hsp70-containing extracellular vesicles could cause full-scale antitumor effect in CT-26 cells by activating of adaptive immunity [36]. In our experiment, we also have observed that SK could significant upregulate the expression of Hsp70 in CT26 cells, suggesting Hsp70 might play a critical role in SK-induced immune activation, and further enhanced the efficacy of PD-1 blockade in CT26 tumor-bearing mouse model. Majority of research have demonstrated that ROS production and ER stress both produced DAMPs to induce tumor cells death [10–13, 37]. In our study, we also found that the expression of Hsp70 was induced by increased ROS after SK treatments, indicating that ROS was a potential inducer of CT26 cell death upon treatment with SK. Besides, we have found that silencing of PKM2 in CT26 cells reverts ROS upregulation induced by SK, indicating that increased ROS was induced by SK in a PKM2-depentent manner.

Our research findings those SK improves the effectiveness of PD-1 blockade in colorectal cancer are consistent with those of Wang et al. [20]. In their study, the authors found that SK and BET inhibitor JQ1 which downregulate PD-L1 expression in tumor cells instead of directly blocking the binding between PD-1 and PD-L1 can synergistically inhibit tumor growth by activating ICD, repolarizing TAM2 and inhibiting glycolysis.

In summary, our study shows that the combination of SK and anti-PD-1 mAb is a potential therapeutic strategy for MSS colorectal cancer. SK is a promising drug candidate for cancer immunotherapy. Until now, SK has not been utilized for prevention or clinical therapy due to its similarity with chemotherapeutic drugs, which can potentially result in side effects. Further research efforts should be directed towards developing a carrier that can accurately deliver SK to tumor tissues.

## Conclusions

Shikonin and PD-1 blockade produced synergistic antitumor effects in CT26 tumor-bearing mouse model by upregulating the expression of Hsp70. Our study elucidated the potential role of ‘Shikonin-PKM2-ROS-Hsp70’ axis in the promotion of efficacy of PD-1 blockade, providing a potential strategy and targets for CRC treatments.

## Data Availability

The authors confirm that the data supporting the findings of this study are available within the article.
